# Sedentary behavior and physical activity one year after colorectal cancer diagnosis: results from the ColoCare Study

**DOI:** 10.1007/s11764-025-01756-x

**Published:** 2025-02-22

**Authors:** Richard H. Viskochil, Tengda Lin, Biljana Gigic, Caroline Himbert, Victoria M. Bandera, Stephanie Skender, Andreana N. Holowatyj, Petra Schrotz-King, Karen Steindorf, Ildiko Strehli, Matthew G. Mutch, Dante Chao, Adetunji T. Toriola, David Shibata, Erin M. Siegel, Christopher I. Li, Sheetal Hardikar, Anita R. Peoples, Jane C. Figueiredo, Martin Schneider, Cornelia M. Ulrich, Jennifer Ose

**Affiliations:** 1 Huntsman Cancer Institute, Salt Lake City, UT USA; 2 University of Massachusetts Boston, Boston, MA USA; 3 Department of Population Health Sciences, University of Utah, Salt Lake City, UT USA; 4 Department of General, Visceral, and Transplantation Surgery, Heidelberg University Hospital, Heidelberg, Germany; 5 Department of Epidemiology, Harvard School of Public Health, Boston, MA USA; 6 Clinical and Translational Epidemiology Unit, Massachusetts General Hospital, Boston, MA USA; 7 National Center for Tumor Diseases , (NCT), Heidelberg, Germany; 8 Division of Preventive Oncology, German Cancer Research Center (DKFZ), Heidelberg, Germany; 9 Department of Medicine, Vanderbilt University Medical Center, Nashville, TN USA; 10 Vanderbilt-Ingram Cancer Center, Nashville, TN USA; 11 Division of Physical Activity, Prevention and Cancer, National Center for Tumor Diseases (NCT) and German Cancer Research Center (DKFZ), Heidelberg, Germany; 12 Department of Surgery, Washington University School of Medicine , St. Louis, MO USA; 13 Siteman Cancer Center, Washington University School of Medicine, St. Louis, MO USA; 14 Division of Public Health Science, Department of Surgery, Washington University School of Medicine and Siteman Cancer Center St. Louis, St. Louis, MO USA; 15 Department of Surgery, University of Tennessee Health Science Center, Memphis, TN USA; 16 Cancer Epidemiology Program, H. Lee Moffitt Cancer Center and Research Institute, Tampa, FL USA; 17 Public Health Sciences Division, Fred Hutchinson Cancer Research Center, Seattle, WA USA; 18 Cedars-Sinai Medical Center, Samuel Oschin Comprehensive Cancer Institute, Los Angeles, CA USA; 19 Department of General, Visceral, Thoracic, Transplantation and Pediatric Surgery, Giessen University Hospital, Giessen, Germany; 20 Department of Media, Information and Design, University of Applied Sciences and the Arts, Hannover, Germany

**Keywords:** Accelerometer, Aging, Objective measurement, Sex differences

## Abstract

**Purpose:**

Physical activity plays key roles in colorectal cancer survivorship; however, the impact of different clinicodemographic outcomes on cross-sectional and longitudinal objectively measured physical activity 12 and 24 months post-diagnosis are unclear.

**Methods:**

ColoCare study participants (*n* = 165) wore an Actigraph GT3x accelerometer for 4–10 consecutive days to objectively assess activity levels 12 and 24 months after colorectal cancer diagnosis and resection. Associations between these clinical/demographic exposures and physical activity outcomes and longitudinal changes were determined using *t*-test, ANOVA *F*-test, and linear regression modeling, adjusting for common confounders (e.g., sex, age, stage).

**Results:**

Key physical activity and sedentary behavior variables significantly differed by demographic status, including minutes of weekly exercise by sex and age (age < 50: 364 min ± 303 min; age 50–70: 232 min ± 263 min; age > 70: 93 min ± 135 min, *p* < 0.001) and (%) daily sedentary time by age (age < 50: 64 ± 10%; age 50–70: 67 ± 7%; age > 70: 71 ± 7%, *p* = 0.003). Within the multivariate model, age was the primary measure consistently associated with activity differences. Participants who wore accelerometers 12- and 24-month post-resection (*n* = 52) significantly increased weekly exercise minutes (214 min ± 208 min vs. 288 min ± 316 min, *p* = 0.04).

**Conclusion:**

Age is the primary clinicodemographic determinant separating physical activity levels in colorectal cancer survivors, and increases in exercise from 12 to 24 months are likely due to consolidation of sporadic daily physical activity into bouts of exercise.

**Implications for Cancer Survivors:**

Colorectal cancer survivors experience different volumes and changes in accelerometer-derived physical activity based on some (e.g., age) but not all (e.g., stage) clinicodemographic variables.

**Supplementary Information:**

The online version contains supplementary material available at 10.1007/s11764-025-01756-x.

## Introduction

Each year over 147,000 Americans are diagnosed with colorectal cancer, the third most common cause of cancer death for both men and women in the USA [1]. Modifiable risk factors contribute to over half of colorectal cancer cases and deaths [2], and the volumes and patterns of post-diagnosis physical activity (PA) have been frequently explored in colorectal cancer survivors, both independently [3, 4] and in conjunction with body fatness [5, 6]. Colorectal cancer survivors report doing less PA compared to cancer-free adults [7, 8], and high amounts of self-reported PA contribute to both a reduced risk of colorectal cancer recurrence and improvements in health-related quality of life [9–11]. Sedentary behavior (SB) has also been evaluated in the context of colorectal cancer risk [12]; however, associations between SB and colorectal cancer survivorship outcomes are less clear. This is potentially due to challenges associated with assessing self-reported SB, which relies on surrogate measures (e.g., television viewing time) that are prone to bias and fail to capture substantial periods of sedentary time, especially in younger adults [13]. Despite these limitations, high amounts of inactivity and television viewing time have been positively associated with poor health outcomes in colorectal cancer survivors, including increased risk of mortality [14–16].

The rise of accurate, noninvasive, and affordable accelerometers has allowed for direct and objective measurement of both PA and SB in colorectal cancer survivorship research. Objective measurement of PA frequently supports the associations between self-reported PA and colorectal cancer health outcomes [17–19]; however, there is a lack of consistency between objective and self-reported measures of SB [20, 21]. Additionally, assessments of objective PA and SB in large cohorts of colorectal cancer survivors have almost always occurred at highly variable timepoints along the colorectal cancer survivorship continuum with respect to the date of diagnosis and/or tumor resection. This results in pooled PA and SB data from patients that can range from 6 months to multiple years after diagnosis, limiting further understanding of how specific clinicodemographic variables and treatments influence the volume and patterns of objectively measured SB and PA at key, clinically relevant timepoints along the survivorship continuum, such as 12 months post-diagnosis.

The period approximately 12 months after cancer diagnosis is often a critical timepoint for colorectal cancer patients as a window for potential behavior change. During this time, PA and SB levels begin to stabilize following acute treatment [22], and many patients transition their focus away from active treatment and towards a greater emphasis on long-term health and recurrence prevention [23]. A deeper understanding of objectively measured PA and SB in colorectal cancer patients at this timepoint would provide the framework necessary to generate personalized lifestyle interventions designed to counteract the adverse effects of cancer treatment, increase quality of life, and prevent colorectal cancer recurrence. Additionally, the cross-sectional nature of most prior assessments of PA and SB in colorectal cancer survivors precludes the evaluation of longitudinal changes in objectively measured PA and SB. Any patient-initiated changes in PA and SB that may occur naturally over the window between 12 and 24 months after diagnosis have therefore not been well described. As a result, it is unclear if the stabilized PA and SB levels observed 12 months after diagnosis persist, regress to lower levels, or increase as patients move away from the period of active colorectal cancer treatment. Any potential patient-initiated changes in PA and/or SB over this time window may have potent ramifications for interventions and warrant exploration.

The purpose of this study was therefore twofold: (1) to objectively measure and describe PA and SB 12 months after a colorectal cancer diagnosis in the ColoCare Study, an international prospective cohort study of newly diagnosed colorectal cancer patients [24], and evaluate clinicodemographic determinants of PA and SB within this population, and (2) to describe longitudinal changes to PA and SB between 12 and 24 months after colorectal cancer diagnosis in a subset of ColoCare study patients. We expect that colorectal cancer patients will have low levels of PA and high levels of SB 12 months after diagnosis, that levels of PA and SB will differ by several key clinicodemographic features (e.g., treatment status, age, rural location), and that PA and SB levels will remain consistent between 12 and 24 months after colorectal cancer diagnosis.

## Methods

### The ColoCare Study and participant clinicodemographics

The ColoCare Study is an international prospective cohort of > 4,000 newly diagnosed colorectal cancer patients (Clinicaltrials.gov identifier: NCT02328677) [24]. Inclusion criteria include adults age 18–89 with newly-diagnosed stage I–IV colorectal cancer. Data and biospecimen are collected at baseline, 3 months, 6 months, 12 months and 24 months. ColoCare participants are enrolled at one of seven sites: Fred Hutchinson Cancer Center (FHCC, Seattle WA), Moffitt Cancer Center (Tampa, FL), Huntsman Cancer Institute (HCI, Salt Lake City, UT), Cedars-Sinai Hospital (Los Angeles, CA), Washington University (St. Louis, MO), University of Tennessee Health Science Center (Memphis, TN), and the University of Heidelberg (Heidelberg, Germany). Accelerometer distribution and data collection at the 12-month timepoint were made optional for participants in Heidelberg Germany from January 2011 to March 2014 and at all US sites except for the University of Tennessee Health Science Center in December of 2017. Colorectal cancer treatment and participant clinicodemographics were abstracted from electronic medical records at each respective ColoCare site and consolidated in a centralized virtual data warehouse at HCI. Demographic information included sex, age, race, ethnicity, body mass index (BMI), and rural/urban location. Clinical information abstracted included surgery/resection status, tumor location, tumor stage at diagnosis, tumor site, and receipt of neoadjuvant and/or adjuvant chemo- or radio-chemotherapy. All patients enrolled in the ColoCare Study provide written informed consent prior to participation and the study has been approved by institutional review boards at all ColoCare data collection sites from study initiation to the present.

### Accelerometer protocol

ColoCare participants wore the Actigraph GT3x accelerometer (Pensacola, FL) for a 4–10-day period, with the option to remove the monitor during nightly periods of sleep. Participants from the Heidelberg, Germany, ColoCare site, wore the monitor around the chest, while those who were enrolled at sites in the USA wore the accelerometer on the wrist. These locations were chosen to minimize the discomfort associated with wear around the waist following surgery and potential interference with stomas/ostomy bags. Duration of monitor wear within this population has been previously validated [25], and detailed methodology of data collection (e.g., sampling frequency) has been previously published [25–27]. Briefly, accelerometers were considered valid if participants wore them for 10 + h/day on 4 consecutive days that included at least one weekend day. Data were analyzed using the Actilife software package (v. 16.3.1 Pensacola, FL). Valid wear time was evaluated using the algorithm from Choi and colleagues [28], and accelerometer counts were converted to PA, SB, and energy expenditure using activity cut points described by Freedson and colleagues [29]. Given the potential risk of wrist accelerometry to misclassify repetitive hand activity (e.g., typing, hammering) as moderate to vigorous physical activity, all participant data was manually visually interrogated for potential misclassification of PA. Periods deemed highly likely to represent misclassified PA were evaluated, and participants considered outliers based on interquartile ranges of key physical activity variables (e.g., weekly exercise minutes, steps/day) were excluded from analysis.

### Accelerometer outcomes

The primary outcomes of interest for this study were the total and percentage of daily time spent sedentary, in light intensity physical activity (LIPA), moderate to vigorous intensity physical activity (MVPA), exercise minutes (MVPA in bouts of > 10 consecutive minutes) and number of exercise bouts per day, number of daily sedentary bouts and sedentary breaks per day, percent of MVPA in exercise, caloric (kcal), and energy expenditure (metabolic equivalents, METS). Additionally, further assessment of exercise minutes was performed to determine whether each participant was meeting the PA guidelines established by the US Department of Health and Human Services (150 min of exercise per week).

### Longitudinal accelerometer collection

Longitudinal data collection of accelerometers is currently occurring at all ColoCare sites; however, at the time of data analysis, only the ColoCare site at HCI was actively collecting and analyzing longitudinal accelerometer data. Collection and analysis of 24-month accelerometer data was identical to collection at the 12-month accelerometer timepoint, and all participants who chose to be involved in the assessment of longitudinal changes to PA and SB were also included in the analysis of 12-month accelerometer outcomes.

### Statistical analysis

All measures of PA and SB were considered continuous except for meeting the PA guidelines (yes/no). Comparisons between patients stratified by clinicodemographic variable (such as surgery, receipt of neoadjuvant chemotherapy, receipt of adjuvant chemotherapy, tumor stage, tumor site, body mass index (BMI), age, sex, urban/rural residence, race/ethnicity) were computed using *t*-tests for exposures that contained only binary variables (e.g., sex) or analysis of variance (ANOVA) *F*-tests for exposures that contained multiple categories (e.g., tumor stage) where appropriate. Further interrogation of significant differences observed within each exposure was addressed using linear regression models, adjusting each model for potential confounding factors such as age, sex and stage. Finally, longitudinal changes to PA and SB variables between 12 and 24 months were determined using paired *t*-tests, and all statistical testing was done using the SAS statistical package (version 9.4) with an accepted a priori significance level of *α* < 0.05.

## Results

### Sample selection and participant characteristics

Valid accelerometer data collected between 1/1/2011 and 8/31/2022 from all sites was initially considered for inclusion in this analysis (*n* = 509). Single accelerometer collections at only the 12-month timepoint from all sites (*n* = 254) were included for initial analysis. Given the focus on the 12-month timepoint as a key window for potential behavior change, we included *n* = 168 accelerometers that were collected within a ± 30 day window of 12 months after surgery. Upon calculation of accelerometer variables within this cohort, *n* = 3 participants were subsequently identified as outliers (MVPA levels > 3 standard deviations greater than the mean), likely a result of misclassification of repetitive wrist movements, and thus, the final 12-month accelerometer cohort included *n* = 165 participants. Of those *n* = 165 participants, an additional *n* = 52 participants from the HCI ColoCare site had valid accelerometer wear data at the 24-month timepoint and thus comprised the longitudinal cohort.

Participant characteristics for the entire cohort (USA and Heidelberg, Germany, *n* = 165) as well as the cohort from the USA alone (*n* = 135) and longitudinal cohort (n = 52) are included in Table 1. There were significant differences between all PA and SB measures when participants from Heidelberg (who wore the accelerometer around the chest) were compared to participants from the USA (who wore the accelerometer around the wrist). Because this represents a true differential measurement of the PA/SB outcomes, participants from the Heidelberg ColoCare site were excluded from the subsequent determinant analysis, and the associations between clinicodemographic exposures and PA/SB outcomes were performed on ColoCare participants from the USA alone (*n* = 135). There were no significant differences regarding clinical and demographic variables between the ColoCare US cohort (*n* = 135) and the overall cohort (*n* = 165), nor were there significant differences between the ColoCare US cohort (*n* = 135) and the longitudinal cohort (with measurements available at 12 and 24 months: *n* = 52).

**Table 1:** Participant characteristics of the US and full* (USA plus Heidelberg, Germany) ColoCare accelerometer cohorts, as well as the cohort of participants who wore the accelerometer at the 12- and 24-month timepoints (longitudinal cohort)

	US cohort (*n* = 135)	Full* cohort (*n* = 165)	Longitudinal cohort (*n* = 52)
Sex (%)			
*Male*	64 (47)	84 (51)	23 (44)
*Female*	71 (53)	81 (49)	29 (56)
Age at diagnosis (n, %)			
Age at diagnosis *(Mean, SD)*	59 ± 14	60 ± 13	59 13
< *50*	31 (23)	33 (20)	11 (21)
*50–70*	73 (54)	93 (56)	30 (58)
> *70*	31 (23)	39 (24)	11 (21)
Race (%)			
*White*	125 (93)	153 (93)	51 (98)
*Black/African American*	5 (4)	5 (3)	0 (0)
*Asian*	2 (1)	2 (1)	0 (0)
*Pacific Islander*	2 (1)	2 (1)	1 (2)
*Other*	0 (0)	2 (1)	0 (0)
Ethnicity (n, %)			
*Non-Hispanic*	127 (94)	157 (95)	52 (100)
*Hispanic*	7 (5)	7 (4)	0 (0)
Body mass index, kg/m^2^ (n, %)			
< *24.9*	24 (18)	37 (22)	10 (19)
*25–29.9*	54 (40)	65 (39)	19 (37)
*30–34.9*	39 (29)	40 (24)	15 (30)
*35–39.9*	11 (8)	12 (7)	4 (7)
> *40*	7 (5)	11 (7)	4 (7)
Rural/urban residence status (n, %)			
*Rural*	48 (36)	48 (29)	16 (31)
*Urban*	84 (62)	84 (51)	36 (69)
Surgery (n, %)			
*Yes*	126 (93)	157 (95)	50 (96)
*No*	9 (7)	9 (5)	2 (4)
Tumor location (n, %)			
*Colon*	83 (61)	97 (59)	29 (56)
*Rectum*	52 (39)	68 (41)	23 (44)
Tumor stage (n, %)			
*I*	18 (13)	22 (13)	9 (17)
*II*	34 (25)	46 (28)	13 (25)
*III*	56 (41)	69 (42)	26 (50)
*IV*	16 (12)	17 (10)	4 (8)
Neoadjuvant chemotherapy (n, %)			
*Yes*	47 (35)	55 (33)	17 (33)
*No*	85 (63)	107 (65)	35 (67)
Adjuvant chemotherapy (n, %)			
*Yes*	59 (44)	73 (44)	16 (31)
*No*	73 (54)	89 (54)	36 (69)

### PA and SB outcomes

PA and SB variables are presented in Table 2. Briefly, the ColoCare participants in this study engaged in high levels of PA including many who were meeting the PA guidelines, including both overall (e.g., steps/day) and exercise-specific PA, and low levels of SB. There were no differences in PA and SB variables between participants from the different sites in the USA.

**Table 2:** PA and SB variables by US, full, and longitudinal ColoCare cohorts

	US cohort (*n* = 135)	Full cohort (*n* = 165)	Longitudinal cohort (*n* = 52)*
Physical activity			
*Light intensity physical activity (min/d)*	280 ± 61	307 ± 81	283 ± 55
*Light intensity physical activity (% wear)*	22 ± 5	21 ± 5	21 ± 4
*Moderate to vigorous physical activity (min/d)*	146 ± 63	180 ± 144	151 ± 54
*Moderate to vigorous physical activity (%wear)*	11 ± 5	10 ± 5	12 ± 4
*Daily exercise^ bouts (n)*	2.5 ± 2.7	2.9 ± 3.6	2.3 ± 2.2
*Daily exercise^ bouts (min)*	33 ± 38	29 ± 36	31 ± 27
*Exercise^ (min/week)*	230 ± 265	206 ± 253	214 ± 208
*Meeting physical activity guidelines*^*#*^* (%)*	67 (50)	74 (45)	28 (53)
*Steps (steps/d)*	8911 ± 3226	8427 ± 3524	9137 ± 2635
*Caloric expenditure (kcal/d)*	956 ± 117	857 ± 561	989 ± 511
Sedentary behavior			
*Sedentary time (min/d)*	876 ± 127	910 ± 128	873 ± 105
*Sedentary time (% wear)*	67 ± 8	69 ± 9	67 ± 7
*Daily sedentary bouts (n)*	16.5 ± 4.3	16.5 ± 4.3	16.7 ± 3.4
*Daily sedentary bouts (min)*	369 ± 102	369 ± 102	364 ± 84
*Daily sedentary breaks (n)*	16.4 ± 4.3	16.4 ± 4.3	16.5 ± 3.4
*Daily sedentary breaks (min)*	958 ± 117	958 ± 117	956 ± 102

*Baseline PA and SB variables within each cohort. ^Moderate to vigorous physical activity in bouts > 10 min. ^#^150 min of exercise/week

### Differences in 12-month PA and SB by determinant/demographic characteristic

We did not observe any significant differences in any PA and SB variables between individuals based on tumor site, receipt of adjuvant chemotherapy, tumor stage, or rural/urban status. We observed significant differences in almost all measures of PA and SB by age (stratified into three age categories, Table 3) and significant differences in exercise-related PA by sex and neoadjuvant treatment status (Online Resource 1), with those individuals who were younger, female, and/or who had received neoadjuvant chemotherapy accruing significantly more PA and less SB compared to older, male, and/or individuals who did not receive neoadjuvant chemotherapy.

**Table 3:** Physical activity and sedentary behavior variables at 12 months post-colorectal cancer resection stratified by age at diagnosis

	Age at diagnosis			
	< 50 (*n* = 31)	50–70 (*n* = 73)	> 70 (*n* = 31)	*p*-value
Physical activity				
*Light intensity physical activity (min/d)*	283 ± 66	278 ± 54	280 ± 70	0.91
*Light intensity physical activity (% wear)*	22 ± 5	22 ± 4	22 ± 5	0.99
*Moderate to vigorous physical activity (min/d)*	182 ± 70	152 ± 54	94 ± 42	< 0.01*
*Moderate to vigorous physical activity (% wear)*	14 ± 5	12 ± 4	7 ± 3	< 0.01*
*Daily exercise^ bouts (n)*	4.0 ± 3.3	2.5 ± 2.5	1.0 ± 1.2	< 0.01*
*Daily exercise^ bouts (min)*	52 ± 43	33 ± 38	13 ± 19	< 0.01*
*Exercise^ (min/week)*	364 ± 303	232 ± 263	93 ± 135	< 0.01*
*Meeting physical activity guidelines*^*#*^* (%)*	23 (74)	38 (52)	6 (19)	< 0.01*
*Steps (steps/d)*	10,441 ± 3572	9157 ± 2920	6799 ± 2468	< 0.01*
*Caloric expenditure (kcal/d)*	1228 ± 731	1024 ± 474	550 ± 302	< 0.01*
Sedentary behavior				
*Sedentary time (min/d)*	849 ± 162	867 ± 113	925 ± 108	0.04*
*Sedentary time (% wear)*	64 ± 10	67 ± 7	71 ± 7	< 0.01*
*Daily sedentary bouts (n)*	15.6 ± 4.5	16.4 ± 4.1	17.7 ± 4.7	0.18
*Daily sedentary bouts (min)*	343 ± 100	368 ± 96	397 ± 112	0.11
*Daily sedentary breaks (n)*	15.6 4.5	16.3 ± 4.1	17.4 ± 4.6	0.25
*Daily sedentary breaks (min)*	1005 ± 120	951 ± 115	928 ± 109	0.02*

^Moderate to vigorous physical activity in bouts > 10 min. ^#^150 min of exercise/week. *Statistically significant difference across each age grouping

**Fig. 1:**
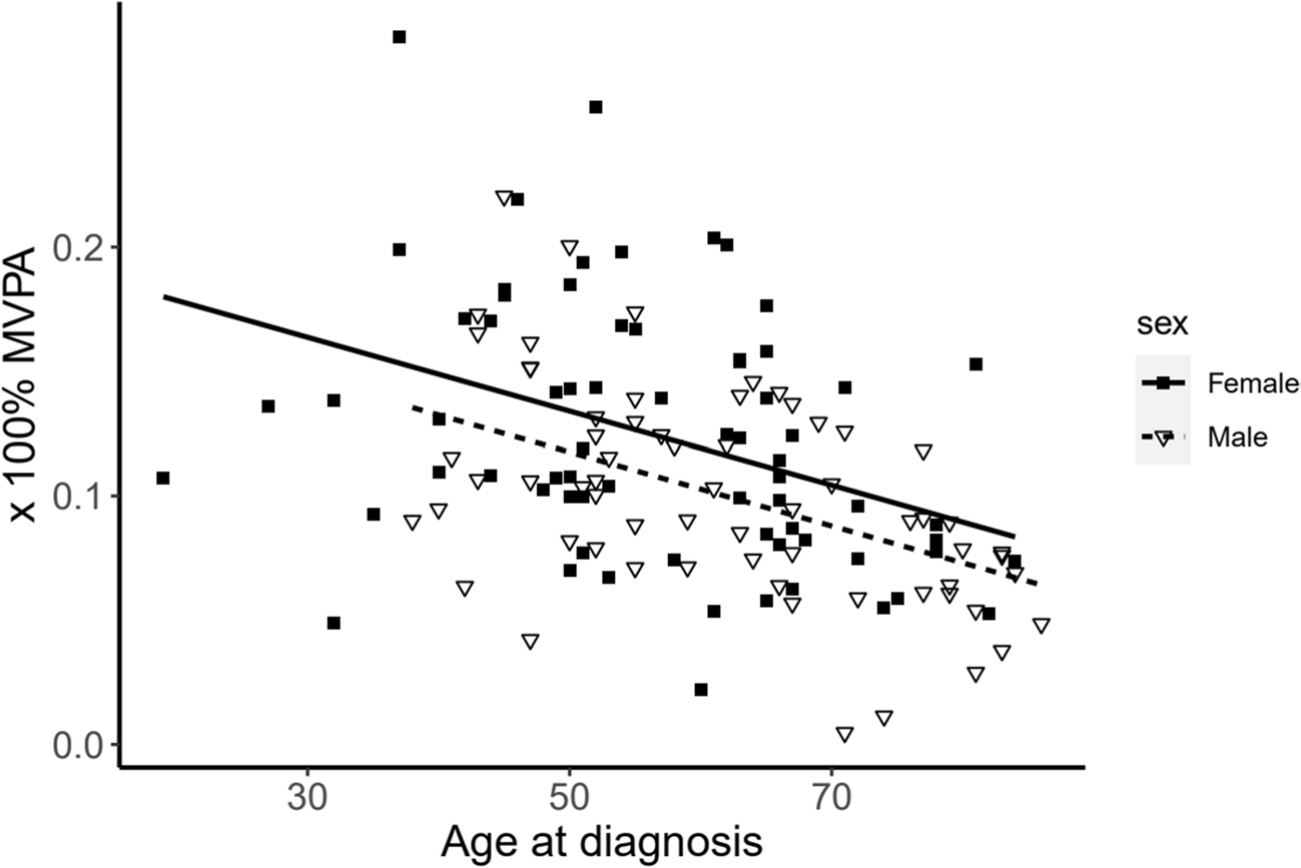
Associations between percent of daily wear comprised of MVPA (%MVPA) and age at diagnosis between men and women. While both variables significantly contribute to %MVPA, age plays a greater role (*β* = − 0.0015; *p* < 0.001) than sex (*β* = 0.016; *p* < 0.033) after adjustment for age and sex

We performed linear regression on outcomes within age, sex, and neoadjuvant chemotherapy status, adjusting each outcome for the other determinants within this analysis that reached significance (e.g., adjusted neoadjuvant status by sex, sex by age). The initial significant difference observed by neoadjuvant chemotherapy status did not persist when adjusted by both sex and age, and the initial sex differences only partially persisted (total minutes of MVPA: *p* = 0.07; minutes of weekly exercise: *p* = 0.05; % of daily wear in MVPA: *p* = 0.03) when adjusted for age. Significant differences by age remained consistent even after adjustment for sex and neoadjuvant chemotherapy status, suggesting that differences in age and sex were primarily leading to the differences in PA and SB observed 12 months after colorectal cancer resection (Fig. 1).

### Longitudinal changes in PA and SB

There were no significant clinicodemographic differences between the cohort of participants evaluated longitudinally and those from the larger cross-sectional cohort of ColoCare participants with 12-month accelerometer data (Table 1). Changes in PA and SB from 12- to 24-month timepoints are presented in Online Resource 2. There were no significant changes in SB or percentage of the day spent in each category of PA; however, energy expenditure was significantly increased at 24 months compared to 12 months. Finally, minutes of weekly exercise significantly increased from 12 (214 ± 208 min/week) to 24 (288 ± 316 min/week) months after colorectal cancer diagnosis (Online Resource 3), despite no change in overall minutes of weekly MVPA, steps, or other measures of PA and a high degree of individual variability.

## Discussion

To date, the quantification of objectively measured PA and SB longitudinally at fixed timepoints along the colorectal cancer survivorship continuum has been limited to a single European cohort [22, 30]. This is the first study to describe accelerometer-derived PA and SB levels 12 months after colorectal cancer diagnosis in patients from the USA, to determine whether any clinicodemographic variables may significantly alter PA and SB at that timepoint, and to quantify PA and SB longitudinally over a 12-month window (from 12 to 24 months post-resection) for changes in these outcomes. This cohort of colorectal cancer patients engaged in relatively high levels of PA compared to other cohorts of colorectal cancer patients whose activity was determined via self-report of leisure-time PA [31–33]. While this may be a result of misclassification of activity or selection bias, it is important to note that the high levels of objectively measured PA are congruent with the high levels of PA assessed via self-report within the ColoCare Study [5, 6], which may reflect the high activity levels of individuals living in Germany and the state of Utah, where a substantial number of ColoCare accelerometer participants reside. Additionally, all participants in the previously mentioned European cohort of colorectal cancer patients with accelerometer-derived PA measurements were meeting the physical activity guidelines at 12 months [22]. This suggests that levels of PA in colorectal cancer patients and survivors may be more complex and internationally variable than previously thought. This is particularly true when PA can be assessed outside of the domain of purely leisure time pursuits by including non-traditional metrics of PA that can be quantified via accelerometers (e.g., work-related PA).

We also observed relatively low levels of SB within this population, equally or slightly lower than the general cancer-free population [34]. This result does not support the findings of Shi and colleagues [21], who observed high levels of sedentary time in colorectal cancer survivors, especially when compared to a cohort of cancer free adults. However, the total minutes of daily sedentary time within each of those cohorts was roughly half the minutes of daily sedentary time observed within the ColoCare Study, suggesting a high discrepancy of percent daily wear that could have contributed to the differing results. Without values expressed as a percent of wear or adjustment for waking vs*.* overnight time, it is unclear if these discordant results represent differences within their respective cohorts’ worth further exploration or if they reflect differences in measurement and/or accelerometer wear protocols. Additionally, there can be challenges in distinguishing between sleep and sedentary time in cancer patients, and without a sleep log, there may be a degree of misclassification in any study investigating SB. Given the relatively small number of studies in which SB is measured via accelerometer in colorectal cancer survivors, it is critical to establish consistent accelerometer wear protocols within this population and to investigate associations between objectively measured SB and survivorship outcomes following a colorectal cancer diagnosis. Future studies designed to evaluate the specific role of daily sedentary time and other metrics of SB (e.g., duration of sedentary time bouts, breaks in sedentary time) on mortality and recurrence in colorectal cancer patients are highly warranted.

We observed significant differences in PA and SB levels when patients were stratified based on age, sex, or receipt of neoadjuvant chemotherapy. However, after adjustment for age, the significant differences based on neoadjuvant chemotherapy were not robust, and differences by sex were substantially reduced. This suggests that age at cancer diagnosis is the primary characteristic that mediates differences in PA and SB levels in colorectal cancer survivors as PA levels stabilize following acute treatment. A significant role of age in PA levels has been consistently observed when evaluating self-reported PA in colorectal cancer survivors [31, 33, 35], with older adults accruing less PA than younger patients. This finding has been confirmed by prior studies to either colorectal cancer or cancer populations alone [18, 22], as it is fairly consistently observed within the general population as well [36]. After adjusting for age, the majority of the sex differences in PA were no longer significant; however, the percent of daily wear time in MVPA remained significant, and both minutes of weekly MVPA and weekly exercise were slightly outside the margin of significance.

While sex differences in PA among colorectal cancer survivors have been previously reported [37, 38], they frequently suggest that men engage in greater volumes of PA when compared to women, unlike our observation that women were more active and less sedentary compared to men. It is unclear why women in the ColoCare cohort may be more active than men; however, it is possible that a greater proportion of women who decide to participate in accelerometer wear are more active and/or are from more active geographical regions (e.g., the intermountain west, [39]) compared to their male counterparts or may experience less occupation-related forced sedentary behaviors. As the ColoCare Study continues to analyze collected accelerometer data, including that which was recently collected in more diverse populations and sites, it is possible that the moderate sex differences in PA that we observed after adjusting for age either fully manifest or disappear completely, which would leave age as the sole demographic characteristic associated with PA and SB 12 months after colorectal cancer diagnosis.

While we observed significant differences in PA and SB when participants were stratified based on several key clinicodemographic features (e.g., age, sex, neoadjuvant chemotherapy), there were no statistically significant differences based on other features that we expected to contribute to differences in PA and SB, including receipt of adjuvant chemotherapy and tumor stage. Adjuvant chemotherapy for the treatment of colorectal cancer is associated with several highly detrimental physiological side effects such as fatigue and reduced quality of life likely limit levels of PA and encourages SB [40]; however, patients who have undergone chemotherapy have also self-reported higher levels of PA in the years following cancer treatment [33]. This suggests that while receipt of chemotherapy may substantially contribute to acute reductions in PA during active treatment [22], it may also provide long-term motivation to increase levels of PA. We did not observe any significant differences in PA based on adjuvant chemotherapy treatment. It is possible that 12 months post-diagnosis may be encapsulated within the transitory window between acute treatment and chronic survivorship, when barriers to engaging in PA are shrinking and motivation to accrue more PA and spend less time sedentary is growing. Advanced tumor stage has also been associated with greater fatigue, reduced quality of life, and more intensive treatment, all outcomes that may reduce PA and increase SB [31, 41]. We did not observe any differences in PA specific to stage at diagnosis; however, this finding is confirmed by several prior studies suggesting no differences in self-reported PA by stage in colorectal cancer survivors [32, 42]. Taken together, the congruency of self-report and objectively measured PA failing to support any differences in PA by stage suggests that the exercise tolerance of advanced stage colorectal cancer patients may be greater than previously thought, and their inclusion into exercise programs should be highly considered.

Finally, we evaluated changes in multiple PA and SB markers longitudinally from 12- to 24-month post-colorectal cancer resection. We observed significant increases in minutes of weekly exercise (MVPA in bouts longer than 10 min) as well as an increase in the percentage of daily MVPA comprised of exercise. Interestingly, this did not occur with an increase in overall MVPA as a result of reductions in sedentary time or light intensity PA. This suggests that participants are consolidating the disparate minutes of MVPA they are already accruing over the course of the day into longer continuous bouts of MVPA that eventually reach the 10-min threshold in which the bout of MVPA can be termed exercise, rather than replacing daily sedentary time with a bout of exercise. While replacing sedentary time with exercise is the preferred way to increase daily levels of MVPA, engaging in longer bouts of continuous MVPA also suggests improvements in general health and fitness that may lead to improved survivorship and reduced all-cause and/or cancer-specific mortality. Multiple interventions have significantly increased exercise volume in colorectal cancer survivors [43–46], including several that have taken place approximately 12–24 months following diagnosis [47–49]. It is often assumed that this increase occurs specifically because of the replacement of sedentary time with exercise. While that may be the primary mechanism, it is also possible that a proportion of this exercise comes as reallocation of daily MVPA, as has been occasionally observed in interventions within the general population [50, 51]. Future studies exploring the allocation of MVPA during exercise training interventions in colorectal cancer survivors may yield more detailed profiles of the true efficacy of interventions to convert sedentary time into activity.

There are several limitations worth consideration. First, accelerometer wear within this cohort was voluntary, and thus, the results may reflect some degree of selection bias, whereas highly active participants were more likely to wear the accelerometers. While this may be a possibility, the high activity levels of the cohort were also reflected in self-report [5, 6], including at baseline, and while we do not have baseline accelerometer data for which to adjust, it is equally likely that the participants in this cohort simply engage in high levels of PA and low levels of SB. The cohort is also racially and ethnically homogenous, with greater than 93% White/Caucasian and 94% Non-Hispanic. Unfortunately, this is highly consistent with other accelerometer-derived cohorts of colorectal cancer patients [17, 22]; many of which match or exceed the racial and ethnic homogeneity of the ColoCare accelerometer cohort. It is critical to develop a greater understanding of PA and SB patterns of diverse colorectal cancer patients from underserved and understudied populations. The ColoCare Study team is actively working to analyze and collect more objectively measured PA and SB in colorectal cancer patients with a focus on ColoCare sites with greater racial and ethnic diversity. We expect that this lack of clarity regarding PA and SB within these populations will be rectified so that all patients may be represented in activity-based research. Due to the sample size, we were unable to investigate clinical outcomes such as mortality and recurrence within this analysis; key survivorship outcomes that may be substantially influenced by levels of objectively measured PA and SB. The associations between patterns of activity and these outcomes are not well-defined; however, the ColoCare Study will present an opportunity to explore these relationships, especially as they pertain to objectively measured SB and colorectal cancer mortality/recurrence, which is otherwise compromised by the use of self-report and has not been thoroughly interrogated in previous studies of colorectal cancer survivorship.

In summary, we observed high levels of objectively measured PA and low levels of objectively measured SB within a large cohort of colorectal cancer survivors 12 months after resection/diagnosis. The primary clinicodemographic variable contributing to differences in PA and SB within this population was age at diagnosis. While adjustment for age removed many of the sex-related differences observed in unadjusted models, it did not completely abrogate this effect, and it is possible that within this cohort women spent significantly more percent of the day in MVPA compared to men. Finally, we observed a significant increase in weekly exercise minutes among a subset of participants who wore the accelerometer longitudinally, at both the 12- and 24-month timepoint. This increase in exercise was not a result of reductions in sedentary time, instead reflecting a greater consolidation of MVPA into bouts greater than 10 min in length.

These results suggest that 12 months after diagnosis represents an opportune window for the implementation of interventions such as personalized exercise training designed to increase PA and/or reduce SB in colorectal cancer patients, as many of the acute factors that may hinder PA (e.g., receipt of adjuvant chemotherapy) have dissipated and patients are beginning to engage in greater volumes and/or more healthy patterns of activity.

## Supplementary Information

Below is the link to the electronic supplementary material.

Supplementary file1 (DOCX 71.7 KB)

## Data Availability

No datasets were generated or analyzed during the current study.
