# Supplementary material for: Sedentary behavior and physical activity one year after colorectal cancer diagnosis: results from the ColoCare Study
Source: J Cancer Surviv. 2025 Feb 22;20(4):1570–80. doi: 10.1007/s11764-025-01756-x (PMC13375754; doi:10.1007/s11764-025-01756-x)
Supplement: Supplementary file 1 — Supplementary file1 (DOCX 71.7 KB) [file 11764_2025_1756_MOESM1_ESM.docx]

**Online Resource 1**: Physical activity and sedentary behavior variables at 12 months post colorectal cancer resection stratified by sex and treatment with neoadjuvant chemotherapy.

|  |  | | |  | **Sex** | |  | | **Neoadjuvant Treatment** | | |
| --- | --- | --- | --- | --- | --- | --- | --- | --- | --- | --- | --- |
|  |  | | | **Male (n=64)** | **Female (n=71)** | | **p-value** | | **Yes (n=47)** | **No (n=85)** | **p-value** |
| **Physical Activity** | |  |  | | |  | |  | |  |  |
|  | *Light intensity physical activity (min/d)* | | | 285 ± 64 | 275 ± 58 | | 0.34 | | 288 ± 54 | 276 ± 63 | 0.29 |
|  | *Light intensity physical activity (% wear)* | | | 22 ± 5 | 21 ± 4 | | 0.71 | | 22 ± 4 | 22 ± 5 | 0.83 |
|  | *Moderate to vigorous physical activity (min/d)* | | | 131 ± 55 | 159 ± 68 | | <0.01* | | 166 ± 67 | 136 ± 59 | 0.01* |
|  | *Moderate to vigorous physical activity (%wear)* | | | 10 ± 4 | 12 ± 5 | | <0.01* | | 13 ± 5 | 11 ± 5 | 0.04* |
|  | *Daily exercise^ bouts (n)* | | | 1.9 ± 2.4 | 3.0 ± 2.9 | | 0.02* | | 3.1 ± 3.2 | 2.2 ± 2.3 | 0.07 |
|  | *Daily exercise^ bouts (min)* | | | 26 ± 33 | 39 ± 41 | | 0.04* | | 40 ± 42 | 29 ± 35 | 0.12 |
|  | *Exercise^ (min/week)* | | | 178 ± 233 | 276 ± 285 | | 0.04* | | 282 ± 295 | 206 ± 248 | 0.12 |
|  | *Meeting physical activity guidelines^#^ (%)* | | | 26 (41) | 41 (58) | | 0.04* | | 29 (62) | 37 (44) | 0.04* |
|  | *Steps (steps/d)* | | | 8,626 ± 2,964 | 9,167 ± 3,446 | | 0.33 | | 9,743 ± 3,154 | 8,520 ± 3,182 | 0.04* |
|  | *Caloric expenditure (kcal/d)* | | | 1,088 ± 613 | 848 ± 493 | | 0.01* | | 987 ± 638 | 956 ± 530 | 0.77 |
| **Sedentary Behavior** | |  |  | | |  | |  | |  |  |
|  | *Sedentary time (min/d)* | | | 898 ± 124 | 857 ± 128 | | 0.06 | | 869 ± 114 | 876 ± 130 | 0.77 |
|  | *Sedentary time (% wear)* | | | 68 ± 8 | 66 ± 8 | | 0.13 | | 66 ± 7 | 68 ± 8 | 0.16 |
|  | *Daily sedentary bouts (n)* | | | 16.5 ± 4.7 | 16.5 ± 4.0 | | 0.97 | | 16.4 ± 3.7 | 16.5 ± 4.7 | 0.97 |
|  | *Daily sedentary bouts (min)* | | | 370 ± 112 | 368 ± 92 | | 0.90 | | 365 ± 88 | 369 ± 109 | 0.84 |
|  | *Daily sedentary breaks (n)* | | | 16.4 ± 4.6 | 16.4 ± 4.0 | | 0.95 | | 16.3 ± 3.6 | 16.4 ± 4.6 | 0.91 |
|  | *Daily sedentary breaks (min)* | | | 967 ± 114 | 950 ± 120 | | 0.45 | | 957 ± 114 | 956 ± 528 | 0.96 |

^Moderate to vigorous physical activity in bouts >10 minutes. ^#^150 minutes of exercise/week. *Statistically significant differences

**Online Resource 2:** Changes in key physical activity variables and sedentary behavior variables from the 12- to the 24-month timepoint.

|  | 12 months | 24 months | Percent change | p-value |
| --- | --- | --- | --- | --- |
| Body Mass Index (kg/m^2^) | 27.6 ± 7.8 | 27.1 ± 6.8 | -1.8 | 0.37 |
| Sedentary Time (% of day) | 66.6 ± 6.7 | 66.7 ± 7.5 | +0.1 | 0.91 |
| Daily LIPA (% of day) | 21.7 ± 3.9 | 21.1 ± 4.1 | -2.8 | 0.20 |
| Daily MVPA (% of day) | 11.7 ± 4.1 | 12.1 ± 5.0 | +3.3 | 0.35 |
| Steps (/day) | 9,136 ± 2,685 | 9,208 ± 2,904 | +0.8 | 0.81 |
| Meeting the PAG (%) | 28 (54) | 30 (58) | +6.6 | 0.35 |
| Sedentary Bouts (/day) | 16.7 ± 3.4 | 16.1 ± 3.6 | -3.6 | 0.31 |
| Breaks in Sedentary Time (/day) | 16.5 ± 3.4 | 15.9 ± 3.4 | +3.6 | 0.32 |
| Energy expenditure (kcal/day) | 989 ± 531 | 1132 ± 553 | +12.6 | **<0.01*** |
| METs | 1.47 ± 0.2 | 1.49 ± 0.2 | +1.3 | 0.34 |
| MVPA as Exercise (%) | 17.3 ± 1.3 | 21.2 ± 1.7 | +18.4 | **0.03*** |

*Significantly different from 12-month timepoint.

**Online Resource 3:** Individual (solid lines) and mean (dashed line) changes in weekly exercise minutes. *MVPA in >10 minute bouts. ^denotes significant difference between the 12- and 24-month timepoint (p<0.05).

**
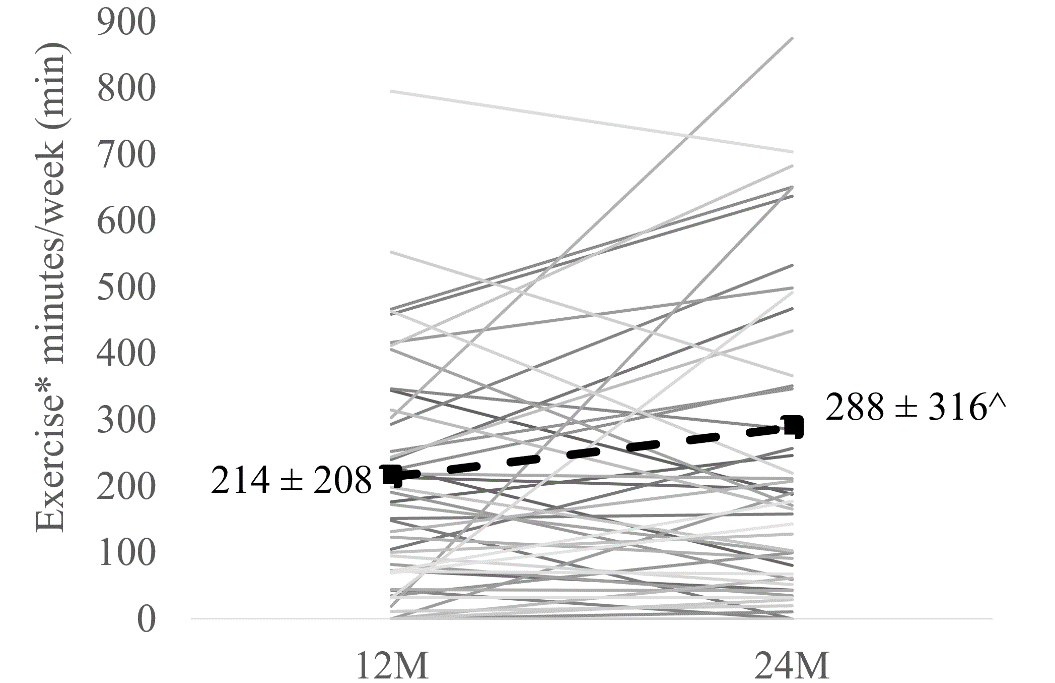
**
